# Supplementary material for: Systematic review and individual-patient-data meta-analysis of non-invasive fibrosis markers for chronic hepatitis B in Africa
Source: Nat Commun. 2023 Jan 3;14:45. doi: 10.1038/s41467-022-35729-w (PMC9810658; doi:10.1038/s41467-022-35729-w)
Supplement: Supplementary file 1 — Supplementary information [file 41467_2022_35729_MOESM1_ESM.pdf]

Supplementary information for: Non-invasive fibrosis markers for chronic hepatitis B in sub-Saharan Africa: a systematic review and meta-analysis

Table of content

Supplementary Figure 1: Flowchart of searches for eligible studies………………………………………………..2

Supplementary Table 1: Characteristics of study sites……………………………………………………………………3

Supplementary Table 2: Risk of bias assessment using the QUADAS-2 criteria………………………..…………4

Supplementary Figure 2: Association of cirrhosis prevalence with age, sex, and reason for hepatitis B testing……6

Supplementary Table 3: Associations with LSM >12.2 kPa (model 1) and LSM >7.9 kPa (model 2) among HEPSANET participants: mixed effects logistic regression model. All p-values are two-sided……7

Supplementary Figure 3: Receiver operating curves for A: APRI (aspartate aminotransferase to platelet ratio index) and B: GPR (gamma glutamyl transferase to platelet ratio) used for the diagnosis of liver stiffness measurement >12.2 kPa (LSM>12.2)……………………………………………………………….....8

Supplementary Table 4: Diagnostic performance characteristics at each site, stratified by reason for testing using APRI with rule-out threshold of 0.65 for the diagnosis of liver stiffness measurement > 12.2 kPa (associated with cirrhosis)……………………………………..…………………………………………..……..9

Supplementary Table 5: Association between participant characteristics and biomarker sensitivity and specificity for the diagnosis of cirrhosis (12.2kPa) with APRI and GPR set at rule-in thresholds: Bayesian bivariate random effects model………………………..………………………………………………….10

Supplementary Figure 4: Diagnostic sensitivity and specificity of APRI for the diagnosis of liver stiffness measurement >12.2kPa (associated with cirrhosis) for subgroups defined by alcohol consumption, body mass index category, sex and reason for screening…………………………..……………..11

Supplementary Figure 5: Sensitivity analyses of rule-in and rule-out thresholds for APRI assessing the use of sex-specific and centre-specific upper limits of normal, and use of an alternative liver stiffness threshold of 9.5 kPa to define cirrhosis……………………………………………………………………………………13

Supplementary Table 6: Sensitivity analysis: Diagnostic performance of APRI and GPR in a subset of 134 patients who underwent liver biopsy as a reference test…………………………………..…………………14

Supplementary Figure 6: Association between liver stiffness measurement and test sensitivity for APRI: Liver stiffness distribution stratified by APRI classification (A & B) and sensitivity of APRI relative to liver stiffness (C & D) among patients with cirrhosis………………………………………..………15

Supplementary Table 7: Search methodology……………………………………………………………………………….16

Supplementary Table 8: List of variables reported by HEPSANET participating sites……………..………17

Supplementary Method 1: Description of bivariate random effects model…………………..……………19

Supplementary Method 2: Validation of APRI model for cirrhosis using 500 bootstrap samples.…..24

Supplementary Figure 1: Flowchart of searches for eligible studies

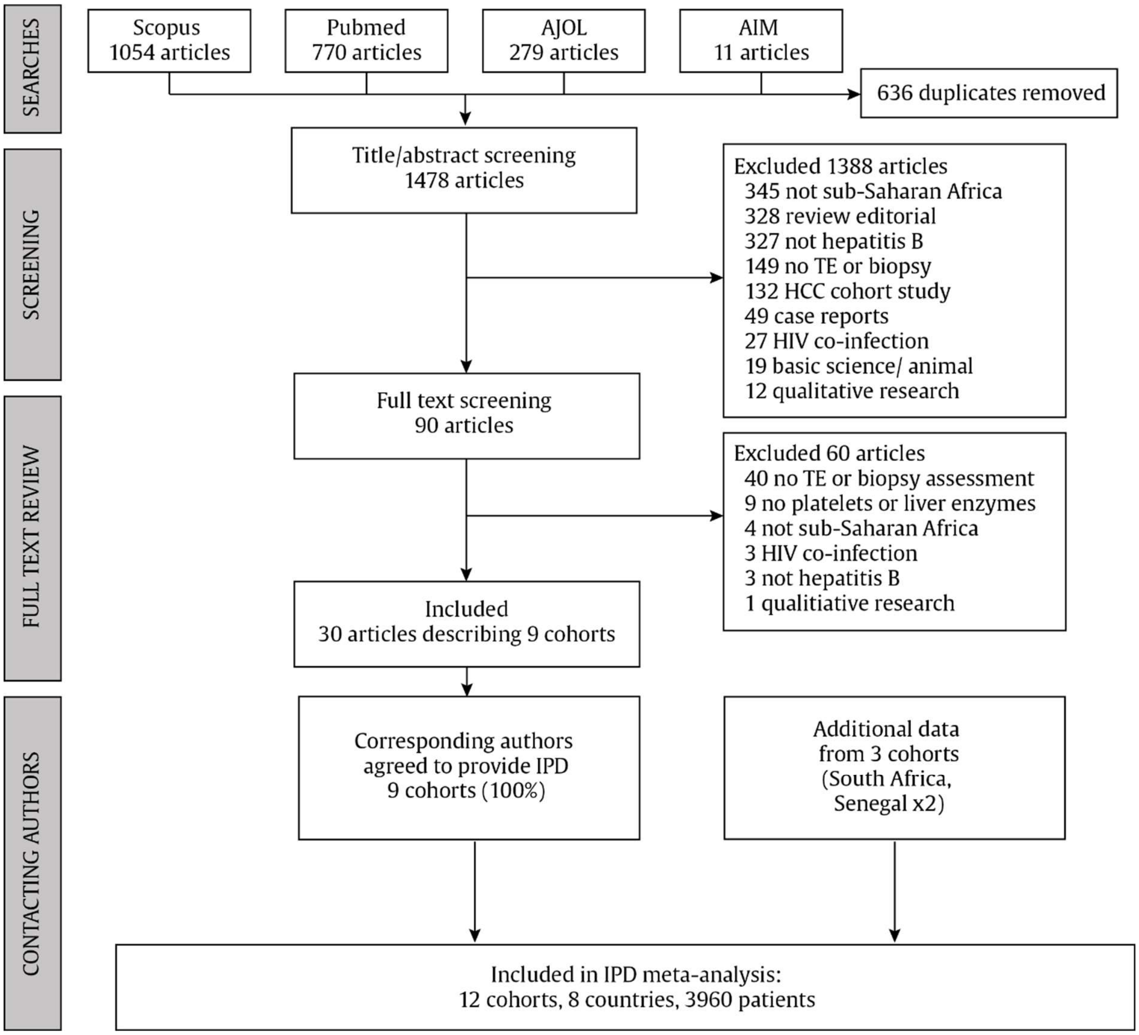

Abbreviations: AJOL, African Journals Online (https://www.ajol.info); AIM, African Index Medicus (https://www.globalindexmedicus.net/biblioteca/aim/); TE, transient elastography; HCC, hepatocellular carcinoma; IPD, individual patient data.

Supplementary Table 1: Characteristics of study sites

Country	Site	Principle investigator(s)	Facility	Year national HBV vaccine introduced	Number of eligible patients	Endemic Schisto-somiasis mansoni	Definition of hazardous alcohol	Biochemistry assay	HBV DNA quantification assay
Ethiopia	Addis Ababa	Desalegn & Johannessen	Referral hospital	2007	1038	No	WHO AUDIT	Humalyzer 3000, Human	HBV Realtime, m2000sp/rt, Abbott & GeneXpert HBV, Cepheid
Gambia	Fajara	Njie & Lemoine	Referral hospital	1990	797	No	>20g/day (none reported)	VITROS 350, Ortho	In-house assay LLQ=50 IU/ml
Senegal	Dakar	Mbaye & Vray	Secondary hospitals (4)	2005	169	No	Not reported	Not reported	Cobas Ampliprep/Taqman v1.0, Roche
Nigeria	Jos	Okeke	Referral hospital	2004	190	Yes	CAGE questionnaire	Cobas, Roche	In-house assay LLQ=20 IU/ml
South Africa	Cape Town	Spearman & Sonderup	Referral hospital	1995	155	No	WHO AUDIT	Coba 6000, Roche	Cobas Amplicor, Roche
Malawi	Blantyre	Stockdale	Referral hospital	2002	97	Yes	WHO AUDIT	AU480, Beckman Coulter	In-house assay LLQ=35 IU/ml[53]
Zambia	Lusaka	Sinkala & Vinikoor	Referral hospital	2005	283	Yes	WHO AUDIT-C	Multiple platforms	In-house; Cobas Ampliprep/Taqman, Roche & GeneXpert HBV, Cepheid
Senegal	Dakar	Fall	Referral hospital	2005	97	No	WHO AUDIT	Cobas 6000, Roche	Cobas Ampliprep/Taqman v1.0, Roche
Senegal	Thies	Lemoine	Referral hospital	2005	300	No	>20g/day (none reported)	VITROS 350, Ortho	HBV Realtime, m2000sp/rt, Abbott
South Africa	Stellenbosch	Maponga	Referral hospital	1990	85	No	Not reported	Architect, Abbott	HBV Realtime, m2000sp/rt, Abbott
Senegal	Dakar	Seydi & Wandeler	Referral hospital	2005	303	No	Not reported	CYNSTART, Cypress Diagnostics, Belgium	COBAS Ampliprep/TaqMan System, Roche
Burkina Faso	Ouagadougou	Sombie	Referral hospital	2005	35	No	Not reported	Architect ci8000, Abbott	HBV Realtime, m2000sp/rt, Abbott

Supplementary Table 2: Risk of bias assessment using the QUADAS-2 criteria

**Country**	**Ethiopia**	**The Gambia**	**Senegal 1**	**Senegal 2**	**Senegal 3**	**Senegal 4**	**South Africa**	**South Africa**	**Nigera**	**Malawi**	**Zambia**	**Burkina Faso**
**Location**	**Addis Ababa**	**Banjul**	**Theiès**	**Dakar**	**Dakar**	**Dakar**	**Cape Town**	**Stellenbosch**	**Jos**	**Blantyre**	**Lusaka**	**Ouagadougou**
**Setting**	**Hospital**	**Community**	**Hospital**	**Hospital**	**Hospital**	**Hospital**	**Hospital**	**Hospital**	**Hospital**	**Hospital/ community**	**Hospital/ community**	**Hospital**
**1. PATIENT SELECTION**												
Was a consecutive or random sample enrolled	Yes	Yes	Yes	Yes	Yes	Yes	No	No[c]	Yes	Yes	Yes	Yes
Was a case-control design avoided?	Yes	Yes	Yes	Yes	Yes	Yes	Yes	Yes	Yes	Yes	Yes	Yes
Did the study avoid inappropriate exclusions?	Yes	Yes	Yes	Yes		Yes	Yes	Yes	Yes	Yes	Yes	Yes
Could the selection of patients have introduced bias?	No	No	No	Yes[a]	Yes[b]	No	Yes	Yes[c]	Yes[d]	Yes[e]	No	Yes[f]
Is there concern that the included patients do not match the review question?	No	No	No	Yes[a]	Yes[b]	No	No	Yes[c]	No	No	No	No
**2. INDEX TESTS**												
Were the index tests interpreted without knowledge of the reference standard?	Yes	Yes	Yes	Yes	Yes	Yes	Yes	Yes	Yes	Yes	Yes	Yes
Could the conduct or interpretation of the index test have introduced bias?	No	No	No	No	No	No	No	No	No	No	No	No
Is there concern the index test, its conduct or interpretation differ from the review question?	No	No	No	No	No	No	No	No	No	No	No	No
**3. REFERENCE TESTS**												
Is the reference standard likely to correctly classify the target condition?	Yes	Yes	Yes	Yes	Yes	Yes	Yes	Yes	Yes	Yes	Yes	Yes

Were the reference standard results interpreted without knowledge of the results of the index test?	Unclear	Unclear	Unclear	Unclear	Unclear	Yes	No	No	Unclear	Yes	Yes	Unclear
Could the reference standard, its conduct, or its interpretation have introduced bias?	No	No	No	No	No	No	No	No	No	No	No	No
**4. FLOW AND TIMING**												
Was there an appropriate interval between index tests and reference standard?	Yes	Yes	Yes	Yes	Yes	Yes	Yes	Yes	Yes	Yes	Yes	Yes
Did all patients receive a reference standard?	Yes	Yes	Yes	Yes	Yes	Yes	Yes	No[c]	Yes	Yes	No	No
Did patients receive the same reference standard?	Yes	Yes	Yes	Yes	Yes	Yes	No	No	Yes	Yes	Yes	No
Were all patients included in the analysis?	Yes	Yes	Yes	Yes	Yes	Yes	No	No	Yes	Yes	No	No
Could the patient flow have introduced bias?	No	No	No	No	No	No	Yes	Yes[c]	No	Yes[g]	Yes[g]	No

[a] Inclusion criteria were inactive HBV carriers with HBV DNA <2000 IU/ml, normal ALT, HBeAg negative.
[b] Inclusion criteria were HBsAg positive for 6 months, treatment naïve, symptom free with HBV DNA >3.2 log_{10} IU/ml.
[c] Subset of patients underwent TE examination at clinicians' discretion- standardised criteria not provided.
[d] Excluded patients with significant alcohol consumption or body mass index >28 kg/m^2
[e] Hospital study recruited patients with suspected cirrhosis based on clinical symptoms or signs suggestive of chronic liver disease.
[f] Only patients undergoing a liver biopsy were included, although this was standard of care at the time for all HBV patients.
[g] Loss to follow up occurred from community diagnosis to treatment eligibility assessment with 94/150 (63%) of HBsAg positive patients being evaluated.
[h] Loss to follow up occurred from referral of patients from the community study to clinical staging at the hospital site with 148/182 (80%) of HBsAg patients having treatment eligibility assessment, of whom 49/148 (33%) had transient elastography.

Supplementary Figure 2: Association of cirrhosis prevalence with age, sex, and reason for hepatitis B testing[a]. Graphs show restricted cubic splines with three knots with respect to age. Shaded areas surrounding central estimates represent 95% confidence intervals.

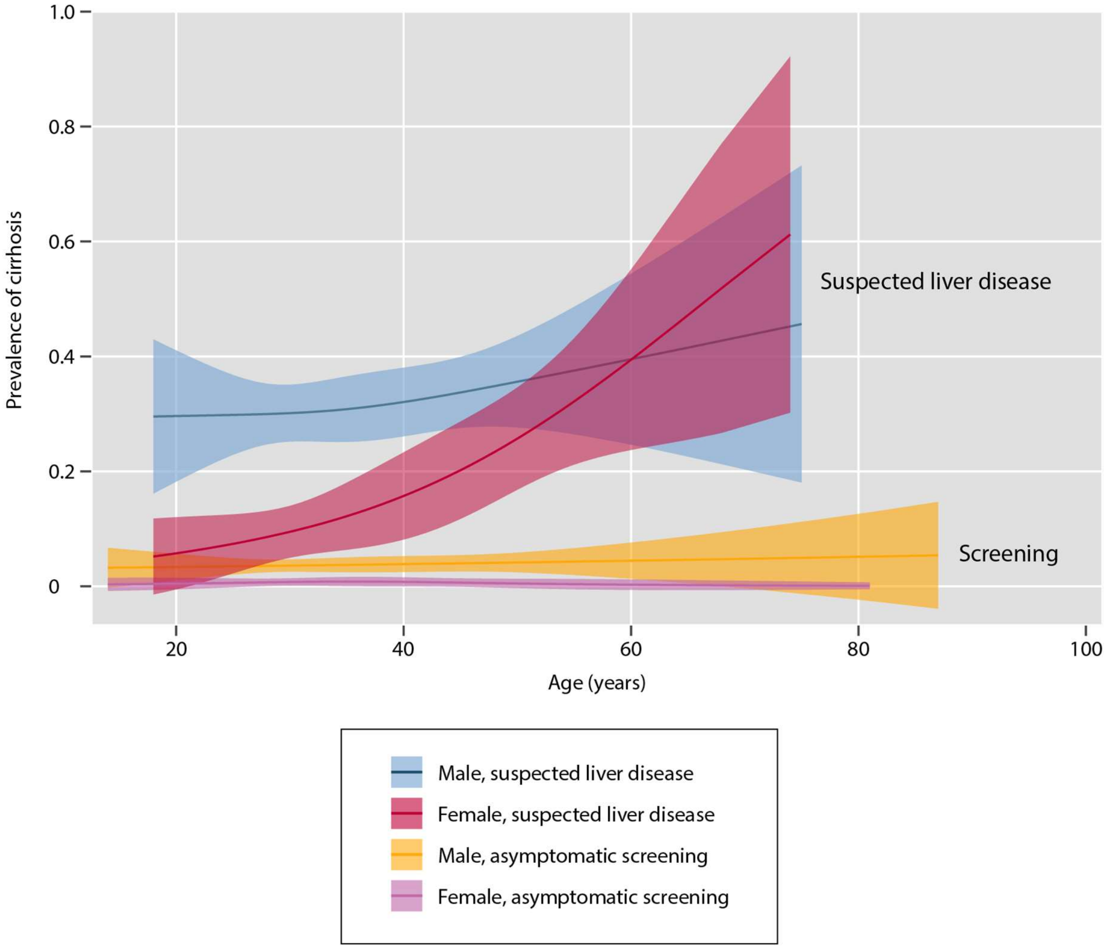

[a] Graphs show restricted cubic splines with three knots with respect to age. Shaded areas surrounding central estimates represent 95% confidence intervals. Source data are provided as a Source Data file.

Supplementary Table 3: Associations with LSM >12.2 kPa (model 1) and LSM >7.9 kPa (model 2) among HEPSANET participants: mixed effects logistic regression model[a]. All p-values are from F tests using Satterthwaite's approximation to degrees of freedom and are two-sided.

Model 1: LSM >12.2 kPa (associated with cirrhosis)

**Variable**	**Univariable association**			**Multivariable model**		
	**Odds ratio**	**(95% CI)**	**P value**	**Odds ratio**	**(95% CI)**	**P value**
Age (per year)	1.03	(1.01 – 1.04)	<0.001	1.03	(1.01 – 1.04)	0.001
Sex (male vs female)	3.53	(2.50 – 4.98)	<0.001	3.27	(2.17 – 4.96)	<0.001
BMI			<0.001			0.06
Underweight	0.95	(0.63 – 1.44)		0.91	(0.57 – 1.47)	
Normal	Reference			Reference		
Overweight	0.46	(0.30 – 0.69)		0.59	(0.37 – 0.94)	
Obese	0.33	(0.14 – 0.78)		0.45	(0.17 – 1.22)	
Suspected liver disease (reference asymptomatic screening)	45.5	(25.7 – 80.5)	<0.001	55.3	(28.0 -109.3)	<0.001

Model 2: LSM >7.9kPa (associated with significant fibrosis)

**Variable**	**Univariable association**			**Multivariable model**		
	**Odds ratio**	**(95% CI)**	**P value**	**Odds ratio**	**(95% CI)**	**P value**
Age (per year)	1.01	(1.00 – 1.02)	0.025	1.01	(1.00 – 1.02)	0.07
Sex (male vs female)	3.37	(2.69 – 4.22)	<0.001	3.40	(2.62 – 4.43)	<0.001
BMI			<0.001			0.005
Underweight	1.00	(0.74 – 1.36)		0.93	(0.66 – 1.30)	
Normal	Reference			Reference		
Overweight	0.49	(0.38 – 0.65)		0.60	(0.44 – 0.81)	
Obese	0.46	(0.28 – 0.76)		0.65	(0.38 – 1.13)	
Suspected liver disease (reference asymptomatic screening)	8.30	(6.32 – 10.89)	<0.001	9.85	(7.11 – 13.7)	<0.0001

[a]Includes random effects for study site.

Supplementary Figure 3: Receiver operating curves for A: APRI (aspartate aminotransferase to platelet ratio index) and B: GPR (gamma glutamyl transferase to platelet ratio) used for the diagnosis of liver stiffness measurement >12.2 kPa (LSM>12.2). The raw ROC curve point estimates are shown as grey dots, connected by straight line segments. The generalised additive model fit is shown as an orange line with the 95% credible interval for the model fits shown as transparent error bands.

A: APRI for diagnosis of LSM>12.2

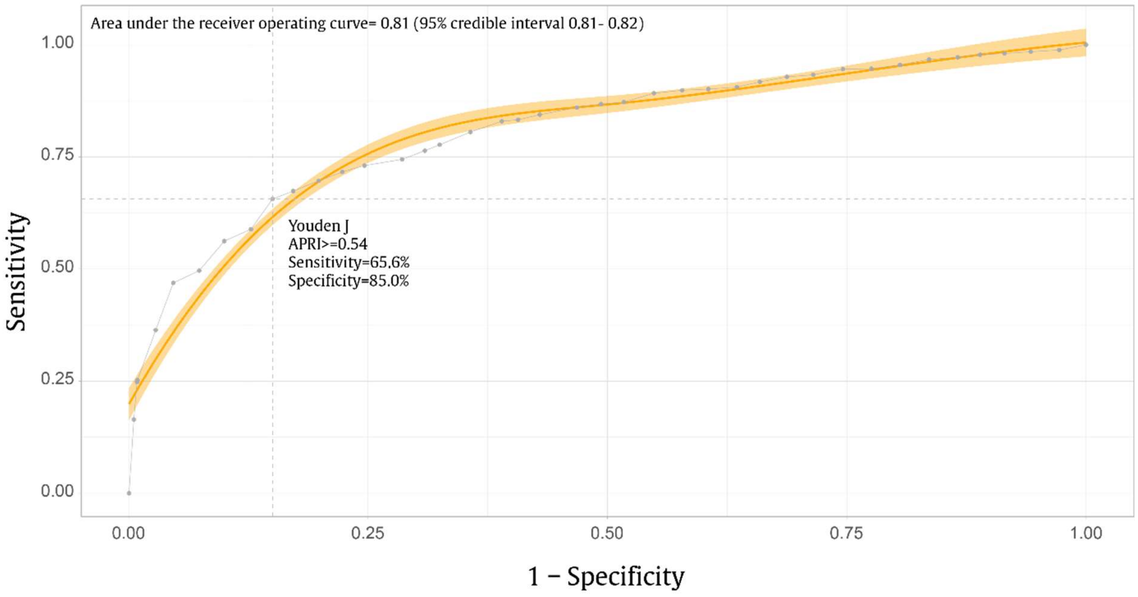

B: GPR for diagnosis of LSM>12.2

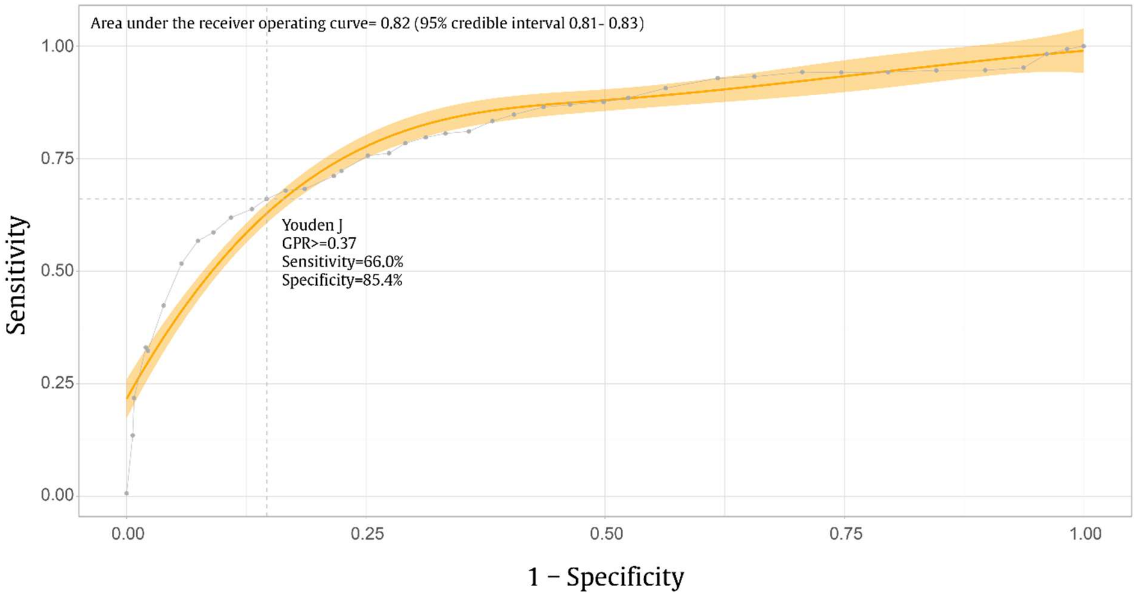

Bayesian bivariate random effects model fitted for different thresholds of APRI using 60 equally spaced quantiles. The ROC curve is a shape-constrained generalized additive model fitted to the raw estimates. Area under the curve is computed from the raw estimates. Source data are provided as a Source Data file.

Supplementary Table 4: Diagnostic performance characteristics at each site, stratified by reason for testing using APRI with rule-out threshold of 0.65 for the diagnosis of liver stiffness measurement > 12.2 kPa (associated with cirrhosis)

Site	Asymptomatic screening populations					Liver disease populations				
	Pr (%)	PPV (%)	NPV (%)	Sensitivity (%)	Specificity (%)	Pr (%)	PPV (%)	NPV (%)	Sensitivity (%)	Specificity (%)
Ethiopia	0.6	0.0 (0-97.5)	99.4 (98.6-99.8)	0.0 (0 - 60.2)	99.9 (99.2-100)	36.5	61.0 (49.6-71.6)	72.0 (65.8 - 77.6)	42.7 (33.6- 52.2)	84.3 (78.6 - 89.0)
Cape Town	3.7	20.0 (2.6-55.6)	98.0 (92.9-99.8)	50.0 (6.8 - 93.2)	92.4 (85.5-96.7)	8.9	16.7 (0.4 - 64.1)	92.3 (79.1 - 98.4)	25.0 (0.6 - 80.6)	87.8 (73.8 - 95.9)
Senegal 1	0	-	100.0	-	96.2 (89.3-99.2)	0.0	-	100		100.0 (39.8 - 100)
Malawi	2.7	25.0 (0.6-80.6)	98.5 (92.0-100)	50.0 (1.3 - 98.7)	95.7 (87.8 - 99.1)	91.7	95.2 (83.3 - 98.8)	50.0 (8.6 -91.4)	95.2 (76.2 - 99.9)	50.0 (1.3 - 98.7)
Nigeria						12.1	48.8 (33.3 - 64.5)	98.6 (95.2 - 99.8)	91.3 (72.0 - 98.9)	86.8 (80.7 - 91.6)
Stellenbosch	11.6	50.0 (11.8-88.2)	96.7 (82.8-99.9)	75.0 (19.4 - 99.4)	90.6 (75.0 - 98.0)					
Zambia	2.1	0.0 (0-14.8)	96.2 (87.0-99.5)	0.0 (0- 84.2)	68.9 (57.1 - 79.2)	54.6	57.1 (18.4 - 90.1)	66.7 (9.4 - 99.2)	80.0 (28.4 - 99.5)	40.0 (5.3 - 85.3)
Gambia	1.3	5.5 (2.2 - 10.9)	99.5 (98.7-99.9)	70.0 (34.8 -93.3)	84.3 (81.6 -86.8)					
Senegal 2	9.5	32.3 (16.7 - 51.4)	96.7 (91.7-99.1)	71.4 (41.9 - 91.6)	84.7 (77.5 - 90.3)					
Burkina Faso										
Senegal 3	6.6	37.5 (18.8 - 59.4)	96.4 (93.3-98.3)	50.0 (26.0 - 74.0)	94.2 (90.6 - 96.7)					
Senegal 4	1.4	20.0 (0.5 - 71.6)	99.1 (96.7-99.9)	33.3 (0.8 - 90.6)	98.2 (95.4- 99.5)	5.9	33.3 (0.8 - 90.6)	95.4 (87.1 - 99.0)	25.0 (0.6 - 80.6)	96.9 (89.2 - 99.6)

Abbreviations APRI, aspartate aminotransferase to platelet ratio index; Pr, Prevalence of cirrhosis; PPV, positive predictive value; NPV, negative predictive value.

Supplementary Table 5: Association between participant characteristics and biomarker sensitivity and specificity for the diagnosis of cirrhosis (12.2kPa) with APRI and GPR set at rule-in thresholds: Bayesian bivariate random effects model[a]

**Biomarker, threshold**	**Sensitivity**	**Specificity**
**Participant characteristics**	**Odds ratio, posterior mean (95% HDI credible interval)**	**Odds ratio (posterior mean) 95% HDI credible interval**
**APRI 0.65**		
Hazardous alcohol consumption	1.19 (0.11 – 2.79)	0.53 (0.24, 0.86)
Underweight	1.41 (0.43 – 2.67)	0.92 (0.57 – 1.33)
Overweight	1.27 (0.31 – 2.63)	1.44 (0.43 – 2.67)
Obese	1.00 (0.00 – 3.10)	2.15 (0.85 – 3.92)
Suspected liver disease	4.96 (0.67 – 1.67)	0.13 (0.06 – 0.21)
Female sex	1.66 (0.53 – 3.17)	2.26 (1.63 – 2.96)
Random effects variance (logit)	0.88 (0.09 – 2.19)	1.27 (0.35, 2.64)
Reference sensitivity/specificity[a]	0.50 (0.32 – 0.68)	0.93 (0.91 – 0.95)
**GPR 0.47**		
Hazardous alcohol consumption	3.00 (0.16 – 8.38)	0.22 (0.07 – 0.41)
Underweight	1.27 (0.24 – 2.66)	1.08 (0.51 – 1.78)
Overweight	1.31 (0.17 – 3.17)	0.57 (0.35 – 0.81)
Obese	0.40 (0.00 – 1.48)	0.72 (0.30 – 1.24)
Suspected liver disease	3.86 (0.39 – 9.85)	0.32 (0.15 – 0.50)
Female sex	0.93 (0.14 – 1.99)	2.64 (1.68 – 3.68)
Random effects variance (logit)	1.27 (0.10 – 3.38)	0.94 (0.20 – 2.10)
Reference sensitivity/specificity[a]	0.60 (0.39 – 0.79)	0.94 (0.91 – 0.96)

Abbreviations: HDI, highest density interval; APRI, Aspartate aminotransferase to platelet ratio index; GPR, gamma glutamyl-transferase to platelet ratio.

[a] Reference category is a male with normal body mass index (18.5-24.9 kg/m^2), without hazardous alcohol consumption, with HBsAg testing conducted for asymptomatic screening. We derived odds ratios (for sensitivity and specificity respectively) for the fixed factors included in the bivariate mixed effects logistic regression model. An odds ratio > 1 indicates that the corresponding covariate, on average, increases the sensitivity (or specificity) and an odds ratio < 1 indicates that the covariate decreases on average the sensitivity (or specificity).

Supplementary Figure 4: Diagnostic sensitivity and specificity of APRI for the diagnosis of liver stiffness measurement >12.2kPa (associated with cirrhosis) for subgroups defined by alcohol consumption, body mass index category, sex and reason for screening. Point estimates at different APRI threshold values are shown as blue (specificity) or orange (sensitivity) dots, connected by straight line segments with 95% credible intervals shown as transparent error bands.

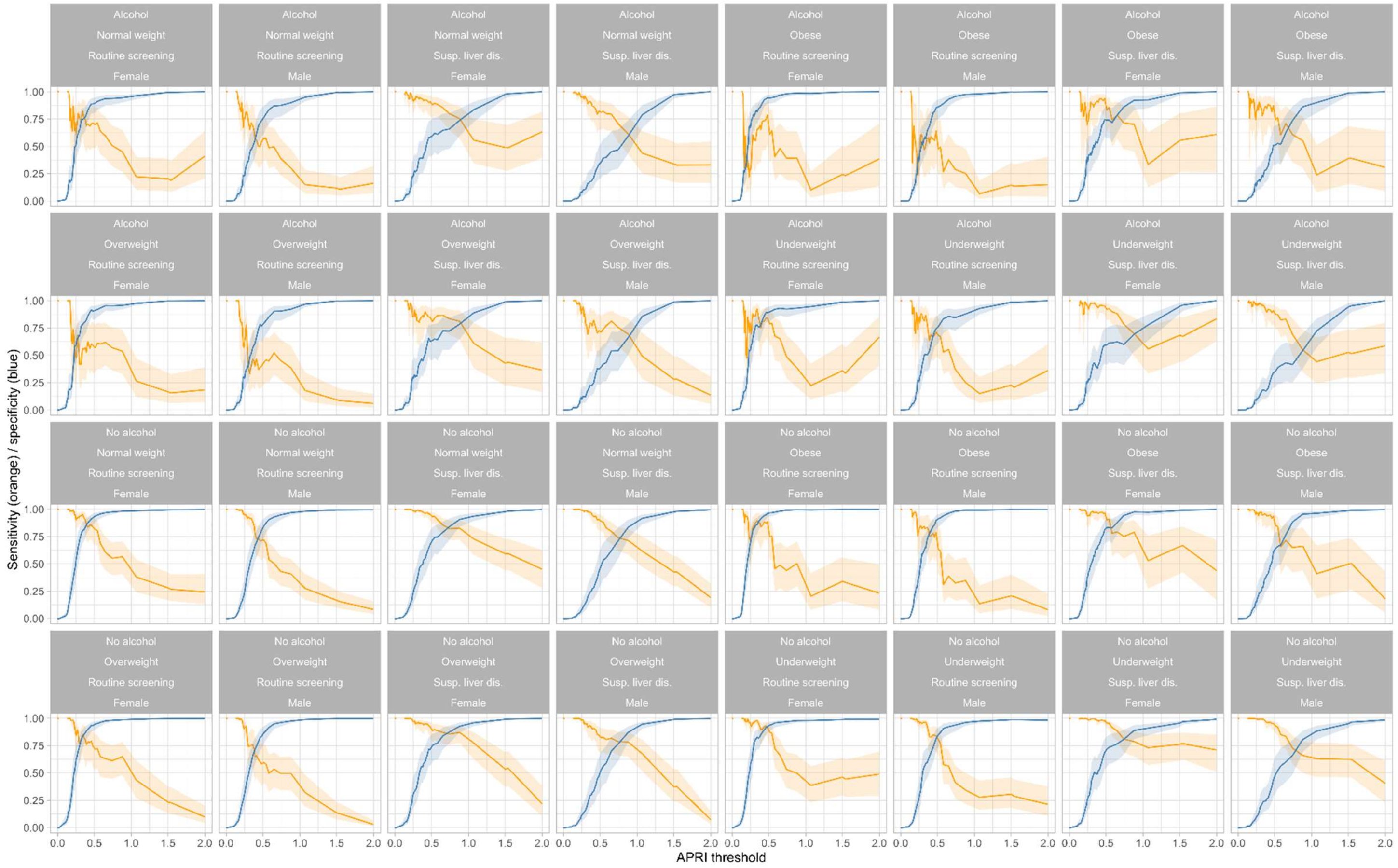

Categories include: hazardous alcohol usage (yes, no), BMI category (underweight, normal weight, overweight, obese), screening reason (suspected liver disease or asymptomatic screening) and sex (male, female). Some subgroups have very few positive cases (LSM>12.2) hence the coefficient estimates for those parameter combinations are characterised by greater uncertainty, resulting in sensitivity curves for some subgroups that are jagged and non-monotonic. The confidence bands are interquartile ranges of the values from the MCMC runs, solid lines are medians computed over the MCMC runs. Source data are provided as a Source Data file.

Supplementary Figure 5: Sensitivity analyses of rule-in and rule-out thresholds for APRI assessing the use of sex-specific and centre-specific upper limits of normal, and use of an alternative liver stiffness threshold of 9.5 kPa to define cirrhosis (n=3548 biologically independent samples). Point estimates are shown as red circles with 95% credible intervals shown as error bars.

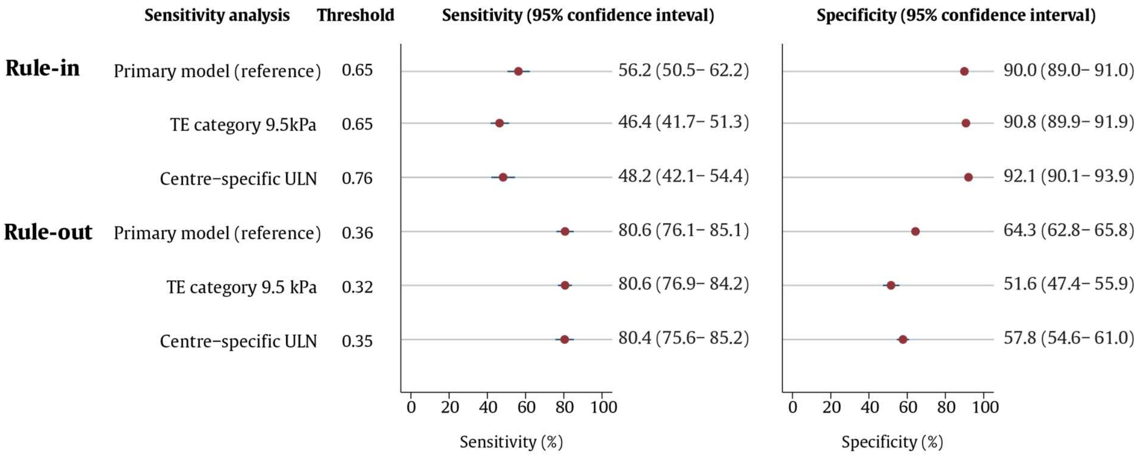

Source data are provided as a Source Data file.

Supplementary Table 6: Sensitivity analysis: Diagnostic performance of APRI and GPR in a subset of 134 patients who underwent liver biopsy as a reference test[a]

Biomarker	Threshold category	Threshold	Sensitivity		Specificity	
Cirrhosis (METAVIR F4)						
APRI	WHO - recommended	2.0	11.1	(0.3 – 48.2)	99.2	(95.6 – 100)
APRI	Rule-in	0.65	100	(66.4 – 100)	73.6	(65.0 – 81.1)
APRI	Rule-out	0.36	100	(66.4 – 100)	36.0	(27.6 -45.1)
GPR	Rule-in	0.47	88.9	(51.8 – 99.7)	78.7	(70.4 – 85.6)
GPR	Rule-out	0.23	100	(66.4 – 100)	40.2	(31.4 – 49.4)
Significant fibrosis (METAVIR F≥2)						
APRI	WHO - recommended	1.5	7.0	(1.5 – 19.1)	97.8	(92.3 – 99.7)
APRI	Rule-in	0.65	48.8	(33.3 – 64.5)	76.9	(66.9 – 85.1)
APRI	Rule-out	0.28	90.7	(77.9 – 97.4)	19.8	(12.2 – 29.4)
GPR	Rule-in	0.40	58.1	(41.1 – 73.0)	77.3	(67.1 – 85.5)
GPR	Rule-out	0.17	93.0	(80.9 – 98.5)	31.8	(22.3 – 42.6)

[a]Among 134 patients who underwent a pre-therapy liver biopsy, 9 had cirrhosis (F4) and 43 had significant fibrosis (F≥2).

Supplementary Figure 6: Association between liver stiffness measurement and test sensitivity for APRI: Liver stiffness distribution stratified by APRI classification (A & B) and sensitivity of APRI relative to liver stiffness (C & D) among patients with cirrhosis. P-values from panels A and B are from Wilcoxon rank-sum tests.

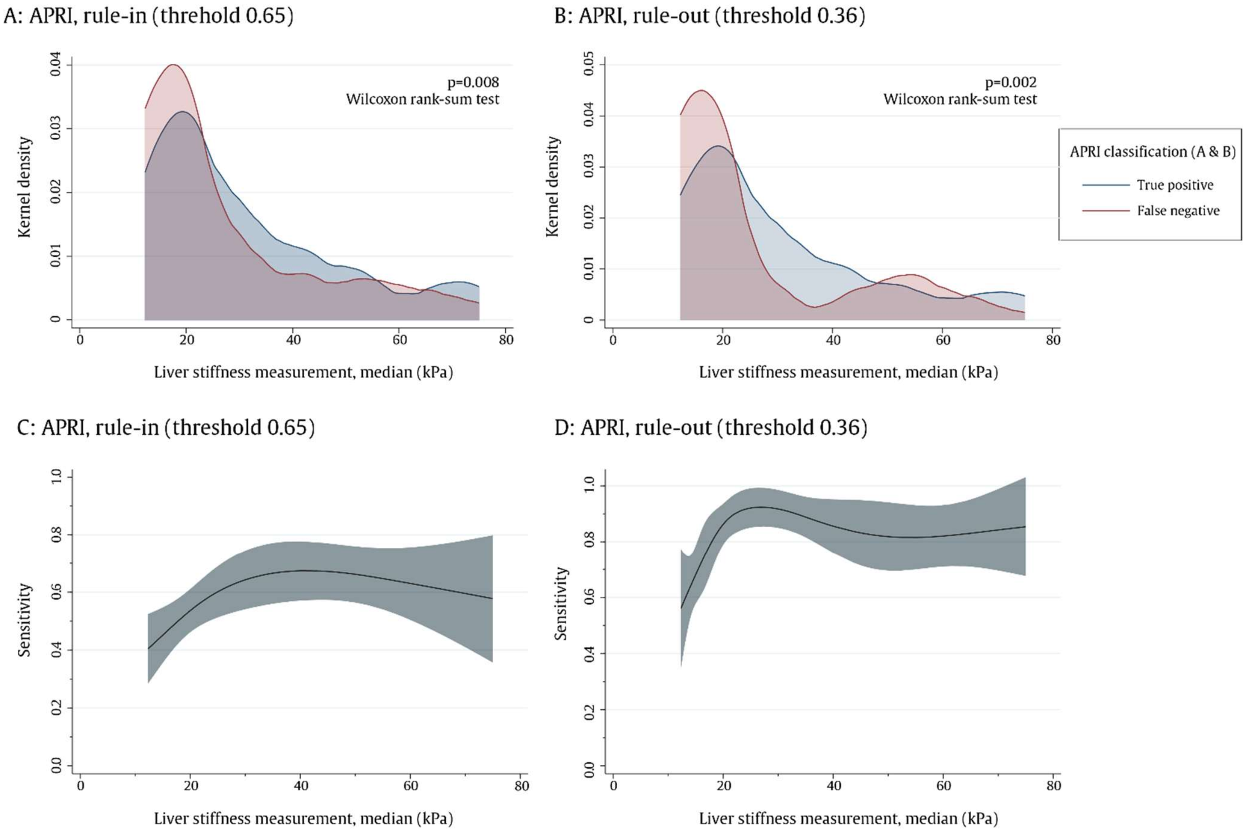

[a] Kernel density plots (A&B) show distribution of median liver stiffness measurements among patients with cirrhosis, stratified by the result of APRI classification at the rule-in (A) and rule-out (B) thresholds. The association between the sensitivity of APRI at rule-in (C) and rule-out (D) thresholds with liver stiffness measurement is shown using a restricted cubic spline with 5 knots, with shaded areas indicating 95% confidence intervals. Source data are provided as a Source Data file.

Supplementary Table 7: Search methodology

Initial search date: 6th October 2020. Search repeated on 12th July 2022. No language or publication date restrictions applied

PUBMED (https://pubmed.ncbi.nlm.nih.gov/)- **770 results**

("liver cirrhosis"[MeSH] OR "elasticity imaging techniques"[MeSH] OR fibrosis[tiab] OR cirrhosis[tiab] OR elastograph*[tiab] OR fibroscan[tiab] OR biopsy, needle[MeSH] OR "liver biops*"[tiab] OR metavir[tiab])
AND (hepatitis B[MeSH] OR hepatitis b[tiab] OR HBV[tiab] OR HBsAg[tiab])
AND (Africa[MeSH] OR Africa*[tiab] OR Angola[tiab] OR Benin[tiab] OR Botswana[tiab] OR "Burkina Faso"[tiab] OR Burundi[tiab] OR Cameroon[tiab] OR "Cape Verde"[tiab] OR "Central African Republic"[tiab] OR Chad[tiab] OR Comoros[tiab] OR Congo[tiab] OR Djibouti[tiab] OR "Equatorial Guinea"[tiab] OR Eritrea[tiab] OR Ethiopia[tiab] OR Gabon[tiab] OR Gambia[tiab] OR Ghana[tiab] OR Guinea[tiab] OR "Guinea Bissau"[tiab] OR "Ivory Coast"[tiab] OR "Cote d'Ivoire"[tiab] OR Kenya[tiab] OR Lesotho[tiab] OR Liberia[tiab] OR Madagascar[tiab] OR Malawi[tiab] OR Mali[tiab] OR Mauritania[tiab] OR Mauritius[tiab] OR Mozambique[tiab] OR Mocambique[tiab] OR Namibia[tiab] OR Niger[tiab] OR Nigeria[tiab] OR Principe[tiab] OR Reunion[tiab] OR Rwanda[tiab] OR "Sao Tome"[tiab] OR Senegal[tiab] OR Seychelles[tiab] OR "Sierra Leone"[tiab] OR Somalia[tiab] OR "South Africa"[tiab] OR Sudan[tiab] OR Swaziland[tiab] OR Tanzania[tiab] OR Togo[tiab] OR Tunisia[tiab] OR Uganda[tiab] OR Zambia[tiab] OR Zimbabwe[tiab])

SCOPUS (https://www.scopus.com/search/form.uri)- **1054 results**

(TITLE-ABS-KEY (africa* OR angola OR benin OR botswana OR "Burkina Faso" OR burundi OR cameroon OR "Cape Verde" OR "Central African Republic" OR chad OR comoros OR congo OR djibouti OR "Equatorial Guinea" OR eritrea OR ethiopia OR gabon OR gambia OR ghana OR guinea OR "Guinea Bissau" OR "Ivory Coast" OR "Cote d'Ivoire" OR kenya OR lesotho OR liberia OR madagascar OR malawi OR mali OR mauritania OR mauritius OR mozambique OR mocambique OR namibia OR niger OR nigeria OR principe OR reunion OR rwanda OR "Sao Tome" OR senegal OR seychelles OR "Sierra Leone" OR somalia OR "South Africa" OR sudan OR swaziland OR tanzania OR togo OR tunisia OR uganda OR zambia OR zimbabwe))
AND (TITLE-ABS-KEY ("elasticity imaging" OR elastograph* OR fibroscan OR "needle biopsy" OR "liver biops*" OR metavir OR cirrhosis OR fibrosis))
AND (TITLE-ABS-KEY ("hepatitis b" OR hbv OR hbsag))

Africa Index Medicus (https://indexmedicus.afro.who.int/) **- 11 results**

(tw:("elasticity imaging" OR elastograph* OR fibroscan OR "needle biopsy" OR "liver biops*" OR metavir OR cirrhosis OR fibrosis)) AND (tw:("hepatitis b" OR hbv OR hbsag))

Africa Journals Online (https://www.ajol.info/index.php/ajol) - **279 results**

Searched using Google Scholar (https://scholar.google.com/)

site:ajol.info (elastography OR liver biopsy OR fibroscan) AND "hepatitis B"

Supplementary Table 8: List of variables reported by HEPSANET participating sites

2.1 Centre-specific variables

Variable	Description/ criteria
Country/ locale	Facility location
Study design	Community or hospital based
Criteria used for valid Fibroscan result	Centre definition
HBV DNA platform	Details of assay, manufacturer, platform
Biochemistry platform	Details of assay, manufacturer, platform for liver enzyme quantification
Schistosomiasis epidemiology	Describe whether endemic hepatic schistosomiasis (*S. mansoni)*
Schistosomiasis diagnosis	Method of diagnostic evaluation for schistosomiasis among centres with endemic disease
Harmful alcohol definition	Definition used for harmful alcohol consumption

2.2 Patient-specific variables (essential variables highlighted in bold)

Variable	Description/ criteria
Patient age	Unit: years
Sex	Male/female
Pregnancy	Current pregnancy
Transient elastography	Fasting (>2 hours) transient elastography result<br>Unit: kPa
Alanine aminotransferase (ALT)	Unit: U/L
Aspartate aminotransferase (AST)	Unit: U/L
Gamma glutamyltransferase (GGT)	Unit: U/L
Platelets	Unit: $x10^9/L$
Bilirubin	Unit: mg/dL
International normalised ratio	Unit: ratio
Hepatitis B e antigen	Positive/ negative
Hepatitis B DNA	Unit: IU/ml
Hepatitis B genotype	Genotype assigned from sequencing
Anti-hepatitis C antibody	Positive/ negative
Hepatitis C RNA	Positive/ negative
Anti-hepatitis D antibody	Positive/ negative
Hepatitis D RNA	Positive/ negative
Body mass index	Unit: kg/m^2
Reason for testing for hepatitis B	Suspected liver disease, due to clinical features of liver disease, or abnormal liver function tests, or abnormal liver imaging; or asymptomatic screening for antenatal care, or blood donation, or family contact of HBsAg positive individual, or community screening.

Current or past hepatitis B treatment	Comprising tenofovir disoproxil fumarate, tenofovir alafenamide, entecavir, lamivudine, emtricitabine, telbivudine, adefovir, interferon.
Family history of HCC or cirrhosis	First- or second-degree relative with cirrhosis or HCC.
Alcohol abuse	Centre-specific definitions were used.
Type 2 diabetes	Ever diagnosed, or treated for, type 2 diabetes mellitus.
Hypertension	Ever diagnosed, or treated for, hypertension.
Hyperlipidaemia	Ever diagnosed with, or treated for, hyperlipidaemia.
Hepatic schistosomiasis	Evidence of schistosomal liver disease by radiology + a positive serum/stool/urine test (according to centre-specific diagnostics)
HCC	Liver tumour(s) diagnosed by radiology or histology.
Ascites	Past or current evidence of ascites, by clinical examination and/or radiology.
Jaundice	Clinically diagnosed with jaundice by a clinician
Variceal bleeding	Upper GI bleeding where endoscopy confirms oesophageal varices.
Hepatic encephalopathy	Cerebral dysfunction observed and diagnosed as HE by a clinician.

Supplementary Methods 1: Description of bivariate random effects model

To calculate sensitivity and specificity, data were pooled using a single-stage individual patient data (IPD) meta-analysis approach. We used a bivariate Bayesian random-effects meta-analysis model for sensitivity and specificity using patient-level covariates with study-level random effects to account for anticipated variability between sites.[21]

Specifically, let $Y_{i,j}$ be the random variable recording the outcome for participant $j = 1, \ldots, n_i$ in study $i = 1, \ldots, m$ for a specific biomarker X and a specific threshold x_t that are currently considered. $Y_{i,j} = 0$ if $X_{i,j} < x_t$ and $Y_{i,j} = 1$ if $X_{i,j} \geq x_t$.

Let $\text{state}_{i,j}$ be the true disease state (according to reference test result, for example cirrhosis present or absent).

The Bayesian bivariate model for sensitivity and specificity is defined by:

$$\begin{aligned} Y_{i,j} &\sim \text{Bernoulli}(p_{i,j}) \\ \text{logit}(p_{i,j}) &= \begin{cases} \beta^{(1)} + \gamma_1^{(1)} \cdot \text{alcohol}_{i,j} + \gamma_2^{(1)} \cdot \text{sex}_{\text{female}_{i,j}} + \gamma_3^{(1)} \cdot \text{BMI}_{\text{underweight}_{i,j}} + \gamma_4^{(1)} \cdot \text{BMI}_{\text{overweight}_{i,j}} + \gamma_5^{(1)} \cdot \text{BMI}_{\text{obese}_{i,j}} + \gamma_6^{(1)} \cdot \text{test reason}_{\text{susp. liver disease}_{i,j}} + u_{1,i} \\ \qquad \text{if } \text{state}_{i,j} = \text{positive} \\ \beta^{(0)} + \gamma_1^{(0)} \cdot \text{alcohol}_{i,j} + \gamma_2^{(0)} \cdot \text{sex}_{\text{female}_{i,j}} + \gamma_3^{(0)} \cdot \text{BMI}_{\text{underweight}_{i,j}} + \gamma_4^{(0)} \cdot \text{BMI}_{\text{overweight}_{i,j}} + \gamma_5^{(0)} \cdot \text{BMI}_{\text{obese}_{i,j}} + \gamma_6^{(0)} \cdot \text{test reason}_{\text{susp. liver disease}_{i,j}} + u_{0,i} \\ \qquad \text{if } \text{state}_{i,j} = \text{negative} \end{cases} \end{aligned}$$

where

$$p_{i,j} = \begin{cases} P(Y_{i,j} = 1 | \text{state}_{i,j} = \text{positive}) = \text{sensitivity} & \text{if } \text{state}_{i,j} = \text{positive} \\ 1 - P(Y_{i,j} = 0 | \text{state}_{i,j} = \text{negative}) = 1 - \text{specificity} & \text{if } \text{state}_{i,j} = \text{negative} \end{cases}$$

and $u_{1,i}, u_{0,i}$ are study-specific random effects

$$(u_{1,i}, u_{0,i})^T \sim N((0,0)^T, \Omega)$$

with Ω a 2x2 covariance matrix.

Stratified models (stratified on reason for testing) are identical, but do not include the test reason variable.

To summarise, we model sensitivity and specificity using a joint logistic regression model, regressing the logit of the probability of a positive test on alcohol consumption level (hazardous consumption or not), sex (female or male), BMI (underweight, normal weight, overweight or obese) and reason for testing (suspected liver disease or routine/community screening). Reference levels for the categorical variables are indicated in bold in the previous sentence; the reference patient is therefore a normal weight male, screened routinely and a non-hazardous alcohol drinker. This model is a random effects, individual patient level meta-analysis model, including a random factor for study in both the sensitivity and specificity marginal models. It is important to use a joint model, given the trade-off between sensitivity and specificity.

Between-study heterogeneity is captured by the matrix of random effects, Ω. The variances in this matrix are the usual τ^2 between-study heterogeneity statistics (one for the logit of sensitivity, the other for the logit of 1-specificity) reported commonly in meta-analyses. We report τ^2, rather than I^2, the percentage of between-study variance not due to sampling error,

as the between-study variances are direct model parameters and are insensitive to both the number of studies and their precision (Rücker, G., Schwarzer, G., Carpenter, J.R. et al. Undue reliance on I 2 in assessing heterogeneity may mislead. BMC Med Res Methodol 8, 79 (2008). https://doi.org/10.1186/1471-2288-8-79). Given the large numbers of models we fitted, we cannot report the posterior distributions for all between-study heterogeneity parameter estimates (we do report posterior means with 95% credible intervals for the models from Figure 3 in Table A.4.1).

Table A.4.1: Posterior means and 95% credible intervals for the random effects variance parameters for log(sensitivity) and logit(1-specificity).

		LSM > 12.2			LSM > 7.9	
		logit(sensitivity)	logit(1-specificity)		logit(sensitivity)	logit(1-specificity)
**Rule-In**	APRI 0.65	0.88 (0.18,2.70)	1.27 (0.47,3.08)	APRI 0.65	0.94 (0.27,2.49)	1.65 (0.58,4.04)
	GPR 0.47	1.27 (0.22,4.20)	0.94 (0.29,2.54)	GPR 0.40	0.84 (0.22,2.46)	0.80 (0.23,2.30)
	FIB4 1.7	1.04 (0.22,3.12)	1.35 (0.46,3.37)	FIB4 1.7	1.42 (0.43,3.75)	1.41 (0.48,3.59)
	ALT 49	0.58 (0.13,1.83)	1.47 (0.49,3.76)	ALT 46	0.60 (0.17,1.67)	2.25 (0.74,5.77)
**Rule-out**	APRI 0.36	2.75 (0.34,11.26)	0.87 (0.35,2.01)	APRI 0.28	1.19 (0.34,3.17)	1.21 (0.50,2.79)
	GPR 0.23	1.33 (0.23,4.68)	0.81 (0.29,2.03)	GPR 0.17	1.41 (0.35,4.55)	1.04 (0.36,2.89)
	FIB4 0.78	0.51 (0.12,1.59)	0.55 (0.23,1.28)	FIB4 0.64	0.69 (0.19,1.92)	0.62 (0.25,1.42)
	ALT 21	0.99 (0.17,3.49)	1.30 (0.50,3.13)	ALT 21	0.79 (0.22,2.11)	1.32 (0.52,3.10)
**Youden J**	APRI 0.54	1.08 (0.21,3.49)	1.11 (0.42,2.66)	APRI 0.46	1.04 (0.31,2.83)	1.18 (0.45,2.81)
	GPR 0.37	1.45 (0.24,4.72)	0.90 (0.29,2.39)	GPR 0.23	1.00 (0.25,2.93)	0.84 (0.30,2.14)
	FIB4 1.6	1.06 (0.21,3.47)	1.12 (0.39,2.82)	FIB4 1.3	1.08 (0.32,2.97)	0.89 (0.35,2.10)
	ALT 36	0.61 (0.15,1.79)	1.13 (0.39,2.77)	ALT 36	0.81 (0.23,2.22)	1.13 (0.38,2.84)
**WHO**	APRI 2.00	1.11 (0.22,3.60)	0.62 (0.13,2.13)	APRI 1.50	1.18 (0.29,3.53)	0.63 (0.12,2.31)

Prior distributions

Since we use a Bayesian paradigm to fit the model, we need to specify prior distributions for the model parameters. These are mostly weakly informative priors:

$$\beta^{(l)}, \gamma_k^{(l)} \sim N(0, 10^5) \quad l = 0,1;\ k = 1, \ldots, 6$$

For Ω, however, it is difficult to specify a truly vague prior. We used the following choice of prior with a diagonal scale matrix and 2 degrees of freedom (same choice as in Riley et al., Stat Med 2008):

$$\Omega^{-1} \sim \text{Wishart}\left(\begin{pmatrix} 1 & 0 \\ 0 & 1 \end{pmatrix}, 2\right)$$

We then conducted sensitivity analyses (off-diagonal element non-zero and larger degrees of freedom). Posterior distributions of the random effects covariance matrix parameters as well as sensitivities and specificities of the diagnostic thresholds (the parameters of main interest) are largely unaffected by different choices of prior (see below).

In the stratified models, the above model is fitted to the data from each stratum based on testing reason (suspected liver disease or other). For those models, the testing reason terms, i.e. the parameters $\gamma_6^{(l)},\ l = 0,1$, are dropped in the above model specification.

The model further specifies distributions for all variables in the model: $state_{i,j}$ (cirrhosis or significant fibrosis present or absent), alcohol, sex_{female} are assumed to follow Bernoulli distributions and body mass index (BMI) a categorical distribution with 4 levels (i.e. a discrete probability distribution where each level has a probability mass π_k, k=1,2,3,4 so that $\sum_{k=1}^{4} \pi_k = 1$; the four levels here are underweight [BMI < 18.5kg/m^2], normal weight [reference level for model; 18.5 kg/m^2 ≤ BMI < 25 kg/m^2], overweight [25 kg/m^2 ≤ BMI < 30 kg/m^2] and obese [BMI ≥ 30 kg/m^2]). Specifying these distributions allows the Bayesian model to handle missing values in the dataset, assuming an ignorable missingness process: at each MCMC iteration, for the unobserved data values, the model samples from the specified distributions with the corresponding distributional parameters learned from the data. As the model is computationally demanding to fit, we used a grid search with 42 (APRI), 41 (GPR), 41 (ALT) and 43 (FIB4) different threshold values evaluated for each biomarker. We aimed for at least 40 different values per biomarker and the slightly different numbers of thresholds per biomarker results from the fact that for some biomarkers several quantiles have the same value.

MCMC settings

The models were written and fitted using JAGS (v4.3.0). For every model 4 MCMC chains were run, using 2,500 adaptive iterations followed by 6,000 main MCMC iterations. We inspected trace plots for signs of non-convergence of the MCMC chains and also computed potential scale reduction factors and effective sample sizes for all model parameters. Summaries of the posterior distributions for parameters of the APRI model with threshold 0.54 are given in Table A.4.2 below.

Table A.4.2: Summaries of the posterior distributions and MCMC diagnostics for the APRI model with threshold 0.54. Rhat is the Gelman-Rubin potential scale reduction factor.

Parameter	Posterior mean	Std. deviation	2.50%	50%	97.50%	Rhat	Effective sample size
exp($\gamma_1^{(1)}$)	0.7545	0.5702	0.1477	0.6039	2.2141	1.00	7801
exp($\gamma_1^{(0)}$)	0.5954	0.1794	0.3262	0.5678	1.0259	1.00	3646
exp($\gamma_5^{(1)}$)	1.2485	2.1140	0.1012	0.7292	5.4162	1.02	8420
exp($\gamma_5^{(0)}$)	1.7934	0.5682	0.9893	1.6952	3.1976	1.00	8975
exp($\gamma_4^{(1)}$)	0.7171	0.4044	0.2198	0.6292	1.7438	1.00	6475
exp($\gamma_4^{(0)}$)	1.1440	0.1722	0.8434	1.1301	1.5199	1.00	7503
exp($\gamma_3^{(1)}$)	1.7039	0.8387	0.6285	1.5238	3.7978	1.00	8898
exp($\gamma_3^{(0)}$)	0.9831	0.1874	0.6700	0.9648	1.4037	1.00	8468
exp($\gamma_6^{(1)}$)	4.1864	3.2009	1.0352	3.3470	12.4673	1.02	656
exp($\gamma_6^{(0)}$)	0.1675	0.0399	0.1028	0.1630	0.2583	1.00	495
exp($\gamma_2^{(1)}$)	2.0839	1.1197	0.7399	1.8359	4.9647	1.00	7785
exp($\gamma_2^{(0)}$)	2.5598	0.3272	1.9863	2.5347	3.2769	1.00	8533
$\Omega[1,1]$	1.0794	0.9374	0.2124	0.8185	3.4856	1.05	226
$\Omega[2,2]$	1.1107	0.6172	0.4170	0.9594	2.6646	1.00	2327
logit^{-1}($\beta^{(1)}$)	0.6869	0.0815	0.5195	0.6908	0.8341	1.00	796
1-logit^{-1}($\beta^{(0)}$)	0.8736	0.0133	0.8457	0.8743	0.8980	1.00	703

Sensitivity analysis for the choice of prior for the covariance matrix of the random effects:

We show here the results of the sensitivity analysis conducted for APRI with threshold 0.54. In addition to the prior used in our analysis, we also investigated the following 2 prior distributions:

$$\Omega^{-1} \sim \text{Wishart}\left(\begin{pmatrix} 1 & 0.5 \\ 0.5 & 1 \end{pmatrix}, 2\right)$$

and

$$\Omega^{-1} \sim \text{Wishart}\left(\begin{pmatrix} 1 & 0 \\ 0 & 1 \end{pmatrix}, 3\right).$$

Histograms summarising the posterior distributions for the covariance matrix parameters and the diagnostic sensitivity and specificity of APRI with threshold 0.54 are shown on the figure below. No substantial differences in posterior distributions are observed.
We note that the posterior mean and 95% credible interval for variance parameter for the study-level random effect for sensitivity for the prior used in the main analysis are 0.88 (0.13, 2.75), those for specificity are 0.90 (0.29, 2.12) and for the covariance parameter are -0.35 (-1.60, 0.56).

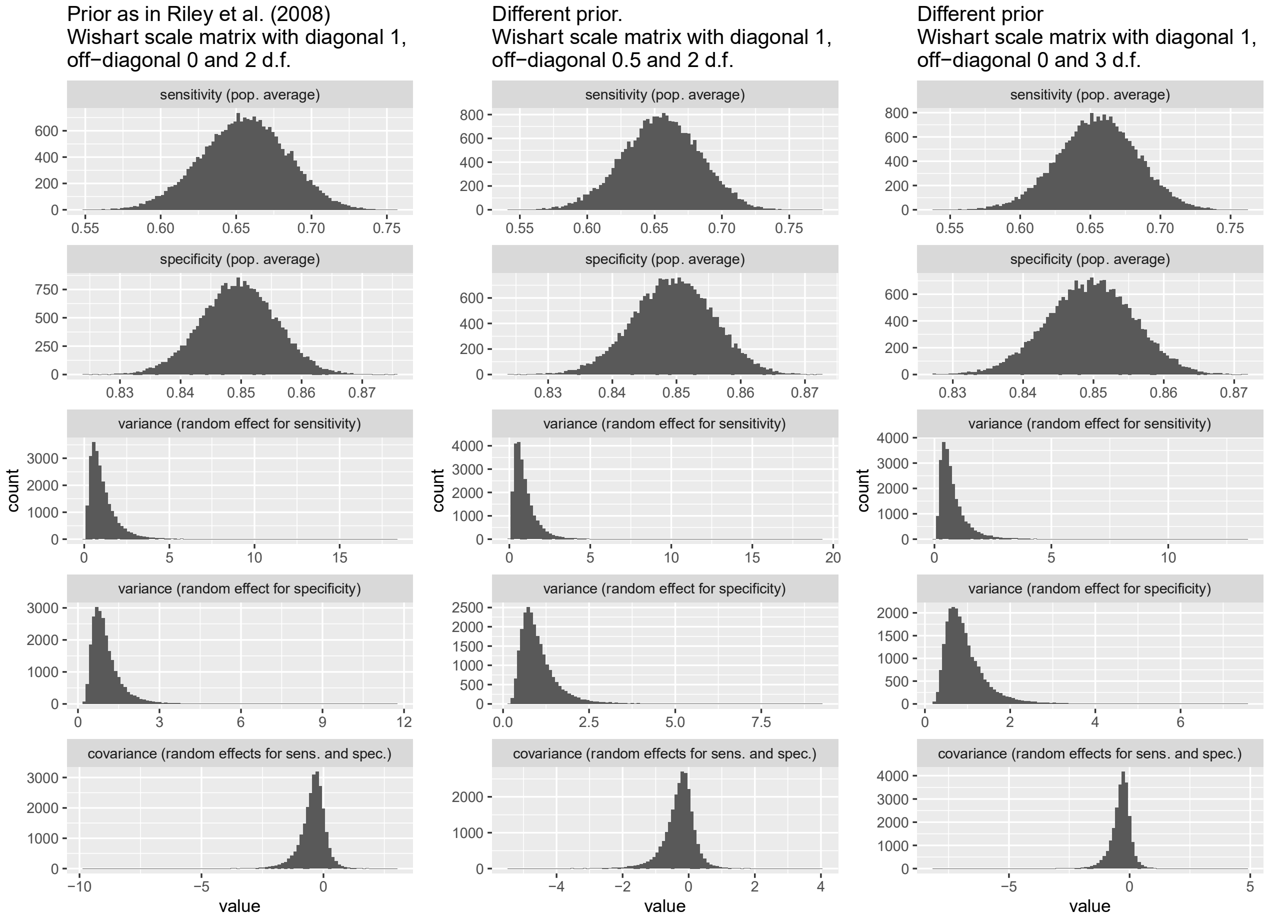

Source data are provided as a Source Data file.

Supplementary Methods 2: Validation of APRI model for cirrhosis using 500 bootstrap samples.

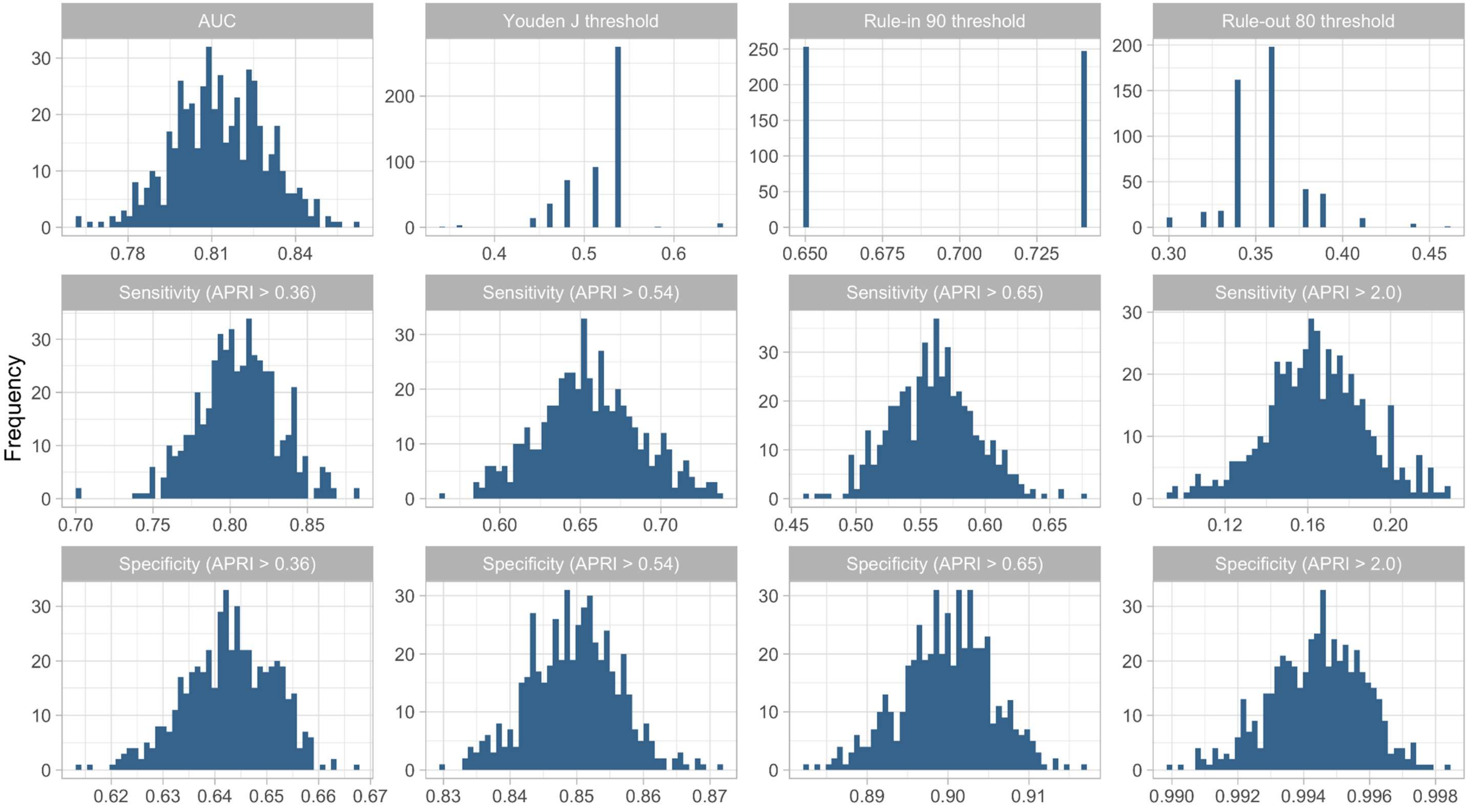

Source data are provided as a Source Data file.
