# Supplementary material for: Systematic review and individual-patient-data meta-analysis of non-invasive fibrosis markers for chronic hepatitis B in Africa
Source: Nat Commun. 2023 Jan 3;14:45. doi: 10.1038/s41467-022-35729-w (PMC9810658; doi:10.1038/s41467-022-35729-w)
Supplement: Supplementary file 3 — Reporting summary [file 41467_2022_35729_MOESM3_ESM.pdf]

nature portfolio

Corresponding author(s): Asgeir Johannessen

Last updated by author(s): Dec 6, 2022

Reporting Summary

Nature Portfolio wishes to improve the reproducibility of the work that we publish. This form provides structure for consistency and transparency in reporting. For further information on Nature Portfolio policies, see our Editorial Policies and the Editorial Policy Checklist.

Statistics

For all statistical analyses, confirm that the following items are present in the figure legend, table legend, main text, or Methods section.

n/a	Confirmed	
☐	☒	The exact sample size (n) for each experimental group/condition, given as a discrete number and unit of measurement
☐	☒	A statement on whether measurements were taken from distinct samples or whether the same sample was measured repeatedly
☐	☒	The statistical test(s) used AND whether they are one- or two-sided<br>*Only common tests should be described solely by name; describe more complex techniques in the Methods section.*
☐	☒	A description of all covariates tested
☐	☒	A description of any assumptions or corrections, such as tests of normality and adjustment for multiple comparisons
☐	☒	A full description of the statistical parameters including central tendency (e.g. means) or other basic estimates (e.g. regression coefficient) AND variation (e.g. standard deviation) or associated estimates of uncertainty (e.g. confidence intervals)
☐	☒	For null hypothesis testing, the test statistic (e.g. F, t, r) with confidence intervals, effect sizes, degrees of freedom and P value noted<br>*Give P values as exact values whenever suitable.*
☐	☒	For Bayesian analysis, information on the choice of priors and Markov chain Monte Carlo settings
☐	☒	For hierarchical and complex designs, identification of the appropriate level for tests and full reporting of outcomes
☒	☐	Estimates of effect sizes (e.g. Cohen's d, Pearson's r), indicating how they were calculated

Our web collection on statistics for biologists contains articles on many of the points above.

Software and code

Policy information about availability of computer code

Data collection	No software was used for data collection.
Data analysis	The QUADAS-2 assessment tool was used to assess the risk of bias. Analyses were conducted in R version 4.1.0 (R Foundation for Statistical Computing, Austria), JAGS 4.3.0, using the rjags package (v4.10),24 and Stata v17 (Statacorp, USA). All R and JAGS code is available from GitHub (https://github.com/gitMarcH/HEPSANET).

For manuscripts utilizing custom algorithms or software that are central to the research but not yet described in published literature, software must be made available to editors and reviewers. We strongly encourage code deposition in a community repository (e.g. GitHub). See the Nature Portfolio guidelines for submitting code & software for further information.

Data

Policy information about availability of data

All manuscripts must include a data availability statement. This statement should provide the following information, where applicable:

- Accession codes, unique identifiers, or web links for publicly available datasets
- A description of any restrictions on data availability
- For clinical datasets or third party data, please ensure that the statement adheres to our policy

We searched PubMed, Scopus, the African Index Medicus, and African Journals Online for relevant articles. The data that supports the findings of this study are available from the corresponding author upon reasonable request.

Human research participants

Policy information about studies involving human research participants and Sex and Gender in Research.

Reporting on sex and gender	Both men and women were represented in this study; 2,133 of 3,548 (60.1%) study participants were men. Sex category was determined based on self-report. We did subgroup analyses for men and women.
Population characteristics	Of 3,548 study participants, the median age was 33 years (interquartile range 28-41) and 60.1% were men. Significant liver fibrosis (liver stiffness >7.9 kPa) was observed in 17.4% and cirrhosis (liver stiffness >12.2 kPa) in 7.3%. Table 1 gives the full clinical demographic description of the study population.
Recruitment	The recruitment of study participants differed between the 12 participating centres, as described under Methods and in Appendix 3 and 7. In >80% of cases (2,824/3,497), the reason for HBsAg testing was asymptomatic screening, whereas 673 individuals (19%) were tested due to suspected liver disease. To minimize selection bias, we performed analyses on the whole dataset, as well as subgroup analyses based on the reason for testing.
Ethics oversight	Ethiopia<br>National Research Ethics Review Committee, Ethiopia (Ref.: 3.10/829/07).<br>Regional Committee for Medical and Health Research Ethics, Norway (Ref.: 2014/1146).<br><br>Malawi<br>National Health Sciences Research Committee of Malawi (Ref.: 16/11/1698 and 15/5/1599).<br>University of Liverpool, UK (Ref.: 1954).<br><br>Zambia<br>University of Zambia Biomedical Research Ethics Committee (Ref.: 013-09-15).<br><br>South Africa<br>University of Cape Town Human Research Ethics Committee, South Africa (Ref.: 667/2020).<br>Stellenbosch University Human Research Ethics Committee, South Africa (Ref.: N17/01/013 and S13/04/072).<br>University of Oxford Tropical Research Ethics Committee, UK (Ref.: OXTREC 01–18).<br><br>Nigeria<br>Jos University Research Ethics Committee, Nigeria (Ref.: JUTH/DCS/ADM/127/XIX/5962).<br><br>The Gambia<br>The Government of The Gambia and Medical Research Council (MRC) Gambia Joint Ethics Committee (Ref.: SCC1266).<br><br>Senegal<br>Senegalese National Health Research Ethics Committee (Ref.: SEN11/34 and SEN19/11).<br><br>Burkina Faso<br>Yalgado Ouédraogo University Hospital Center Institutional Review Board, Burkina Faso (Ref.: 0782).

Note that full information on the approval of the study protocol must also be provided in the manuscript.

Field-specific reporting

Please select the one below that is the best fit for your research. If you are not sure, read the appropriate sections before making your selection.

☒ Life sciences ☐ Behavioural & social sciences ☐ Ecological, evolutionary & environmental sciences

For a reference copy of the document with all sections, see nature.com/documents/nr-reporting-summary-flat.pdf

Life sciences study design

All studies must disclose on these points even when the disclosure is negative.

Sample size	We did not calculate a priori sample size. The priority was to collect as much data as possible from multiple centres and countries in sub-Saharan Africa, to make the findings representative of patients on the continent. With >3,500 patients this is one of the largest IPD meta-analyses in its field, giving precise estimates of diagnostic performance of the biomarkers of interest.
Data exclusions	The procedure for database search and identification of relevant studies is described under Results and Appendix 6. All invited study centres agreed to share individual patient data. Of 3,960 HBsAg-positive patients from eight countries, we excluded 412 participants as described in Figure 2. The reasons for exclusion were: i) current anti-HBV therapy, ii) positive or unknown HIV status, iii) grossly elevated ALT/AST which can interfere with liver stiffness measurement, and iv) positive hepatitis C or D antibodies.

Replication	We validated the model using bootstrap resampling with 500 bootstrap samples obtained from the original dataset for internal validation. We secondly assessed the performance of the new optimised rule-in and rule-out thresholds on a subset of patients who underwent pre-therapy liver biopsy using an alternative reference test, the METAVIR histological fibrosis scores.
Randomization	Not relevant as this was not an interventional study.
Blinding	Not relevant as this was not an interventional study.

Reporting for specific materials, systems and methods

We require information from authors about some types of materials, experimental systems and methods used in many studies. Here, indicate whether each material, system or method listed is relevant to your study. If you are not sure if a list item applies to your research, read the appropriate section before selecting a response.

Materials & experimental systems

n/a	Involved in the study
☒	☐ Antibodies
☒	☐ Eukaryotic cell lines
☒	☐ Palaeontology and archaeology
☒	☐ Animals and other organisms
☒	☐ Clinical data
☒	☐ Dual use research of concern

Methods

n/a	Involved in the study
☒	☐ ChIP-seq
☒	☐ Flow cytometry
☒	☐ MRI-based neuroimaging
